# On the Teaching of Anatomy to Advanced Medical Students

**Published:** 1892-06

**Authors:** 


### SELECTIONS.

### On the Teaching of Anatomy to Advanced Medical Students.

[Reprinted from the Medical News, December 26, 1891].

The importance of anatomy to the physician and surgeon has caused the method of teaching this science to be largely determined by practitioners. The student is taught the elements of histology, the shapes and numbers of organs, the outlines of regions and their mutual relations. Other facts than those named belong in a very remote degree to the needs of practice; and when the great number of medical topics is considered, which is of necessity brought to the attention of the student, it is no wonder that governing bodies are disposed to disregard all phases of instruction that do not have direct claim upon the physician's time and service,

But science is rarely pursued for practical good. The acquisition of knowledge for its own sake—the determination of general principles that reveal the existence of law—awakens and maintains pleasures and interests in the mind of the anatomist compared with which the practical uses that he can make of the knowledge appear to be poor and mean. With as much propriety one might say that navigation is the highest use that can be made of the study of astronomy, as to assert that the chief end of the study of anatomy is to apply its tenets to medicine. These statements are made not to lessen the dignity and importance of practical work, but respectfully to claim that such work does not comprise all the value, indeed scarcely more than a small fraction of the value that pertains to the whole.

In his *New Atlantis*, Lord Bacon says: "We have three of our fellows that bend themselves, looking into the experiments of others, and cast about how to draw out of them things of use and practice for man's life and knowledge, as well for works as for plain demonstration of causes, means of natural divinations, and the easy and clear discovery of the virtues and parts of the bodies. These we call dowry-men or benefactors. Lastly, we have three that raise the former discoveries by experiments into greater observations, axioms and aphorisms. These we call the interpreters of nature."

I hear a response to the foregoing statement that the structure of animals exhibited on a broad scale is already taught to classes in the scientific schools, and that in the scheme of a university education the biological subjects are as well advanced as any others in the curriculum. This is an imperfect, if not misleading, presentation of the facts. It is true that the rudiments of the structure and functions of animals and plants are taught. But to students already advanced by general training and by preliminary work in natural history, little is presented that prepares them to discuss the more intricate problems.

To my mind the scheme of university work is unsatisfactory until opportunity is afforded to men, who, after completing their biological and medical training, may desire to still further advance. Conceding that the question of maintenance has been settled, either by the possession of private means or by endowment of fellowships, what courses of instruction are afforded these advanced men? As a rule, nothing, or next to nothing. It is customary for such novitiates to reside abroad for several years, where amid numerous centers of learning are found one or more masters, the disciples of whom they become. The advantages of travel being considered, it may be said that with the comparatively easy means of obtaining the best instruction the present scheme is, on the whole, adequate. With such a conclusion I cannot agree. If it were true we might in reason have stopped long ago in our lines of university expansion. Independence in intellectual as well as in political life should be the object of American citizenship.

First, and always let us remember that medical investigators are those it is desired to train. It is for men that are already imbued with the desire to pursue their researches in anatomy that I appeal. They stand in this field with what preparations can be given them for usefulness. They are medical biologists—

medical anatomists. They are not restricted to the problem of the relief of suffering, and yet they are occupied with those other problems upon which the true solution of all depends.

For such instruction I would have a specially-designed museum and a specially-equipped laboratory. It may be assumed that in every great medical school, from among the large number of matriculates (men already trained and of the best quality), two or three of the type described will present themselves for an advanced course in anatomy. I am prepared for the objection that this is too large a number. But, so far as I know, no one has attempted to ascertain how many men in each class of graduates would come forward, and my impressions are based upon the number of workers in the general field of biology —some of whom, at least, would have pursued these, or similar studies, had any systematized course been presented to them. I will, therefore, begin with three men a year. To this number may be added as many young teachers, tutors, curators and prosectors who would avail themselves of the instruction. The work might be initiated in either of the halls of biology or of medicine. If the course were well established, it would be well to institute a laboratory and museum distinct from any on the university grounds.

I am of the opinion that the administrative success of such separation of collections would be assured. All must approve of the ethnological collection of Harvard being distinct from the Museum of Comparative Zoology, and of both in turn being set apart from the museum in the Medical School. In like manner I assume that there is no reason why series of specimens arranged in illustration of principles that are not taught either in the preliminary or in the proper medical courses should be necessarily connected with one or the other museum. The collections should be in the main designed to accommodate the preparations that are used in the illustration of general lectures. Museums that teach by the specimens being removed from the cases to the lecture halls are radically distinct from museums that teach by the conservation of series that are arranged and labeled for instruction as they stand, and which should be rarely, if ever, disturbed.

The following, treated in some detail, embrace the topics that occur to me at this time as appropriate subjects for instruction: The study of the human brain; especially the study of the mammalian and avin brains, both of the gross and the minute anatomy, the localization of functions, etc. The study of muscular anomalies and their homologies in the normal myology of the vertebrates. The study of animal locomotion and its application to the morphology of the vertebrate limb, and in general the application of photographic methods in studying animal locomotion. * Studies in craniology, especially the comparative studies of human and mammalian crania. The study of osteological variations, with a similar application to the normal anatomy of the lower animals and the beginning of morbid processes. The study of nutritive processes on tissue as correlated to age.†

In addition, courses of experimental morphology might be essayed. Such investigation could be encouraged without encroaching on the domain of physiology, as the votaries of this science somewhat arbitrarily restrict it. Indeed, much of the study of animal locomotion would be experimental, as would also be the study of protoplasm in viscid media, under rotation, compression, etc. The effects of light, temperature, water in motion and at rest, etc., on organization, would naturally find a place. Experiments on mutilation of embryos might also be undertaken.

Lectures on correlation of structure, on vegetative repetition,

* Instantaneous photographs have given us definite conceptions of the behavior of the manus and pes in terrestrial and aerial movements. I had the honor to point out as the result of a study of the negatives taken by Mr. E. Muybridge under the auspices of the University of Pennsylvania, that the ground is touched by the outer border of the foot and is left by the inner border, and that the impact represented by this transition is expressed by an oblique line that extends from without inward (ecto-entad) across the metapodium. Prof. H. F. Osborne, by studying the carpus and tarsus in extinct forms of mammalian life, has found that this conclusion is of value in studying the evolution of the parts. From this we can conclude that, as a result of a photographic plant in connection with advanced anatomical work, discoveries could with some confidence be anticipated.

† This would form a morphological study on the nature of age, and would more particularly embrace a consideration of the immature and senile forms as compared with the typically adult, as well as the retention of juvenile characters in the adult.

on the relation existing between phyllogenetic and teratological processes, could be given, as well as the study of the laws of heredity, especially in attempting to answer the question of the transmittal of acquired characters.

The teeth are so responsive to the constitutional peculiarities of the individual that their peculiarities can be seen and readily detected. The method of procuring accurate impressions can be applied, and the plans of preserving the form of teeth be easily accomplished.

As is known to the zoologist, the parts involved in the act of mastication are important in the classification of the mammalia, the slightest departure in the form, number, position, and rate of development of the teeth being, for the most part, correlated with other variations in the economy, while the shapes of the lower jaw and of those portions of the skull that afford surfaces for attachment of the masticatory muscles are of importance. No structures of the body resemble the teeth in the character of their response to morbid impressions; no other organs are arranged in progressive series, and none other than these are evolved after birth. Hence the effects of disease and accidents to which the teeth are subjected are sure to be recorded in the shapes of the crowns and roots.

If the student of heredity were to have placed at his disposal a collection of the casts of the permanent teeth of three generations—that is to say, of the parent of the subject, the subject himself, and the children of the subject—and if a clinical history were secured of the diseases and accidents that these persons had incurred, a tenable argument might be established as to the significance of the contrasts or resemblances in the forms of the teeth.

Thus, if three generations were expressed by the letters A, B, C, and if B is the subject of an acquired character (let us say from scarlet fever or measles), the new form of structure seen in the second and third molars may be transmitted to C. But in order to prove this it is necessary to know the peculiarities of these teeth in A. Hence, the teeth of the ancestors and descendants of the person who exhibits the acquired character must be known. A somewhat similar plan of observation could be made on the teeth of the lower animals. It is strange that those teeth with endless pulps in which growth is rapid and interference with their relations causes permanent records to be made in malformation, should not have been used in studies of nutrition.

In connection with myological studies a number of minor problems suggest themselves; such, for example, is the nature of white and red muscles. It has been noted that in ostriches that have been confined in zoological gardens the muscles of the leg undergo fatty degeneration and become white in color; it is also known that the pectoral muscle in many of the gallinæ is white, presumably from the fact that they are used but for short and infrequent flights. How evident is the conclusion that a systematic study of all muscles of active birds living in enforced confinement, as compared with the relatively-active muscles in feral forms, might be undertaken with a fair prospect of throwing light upon the nature of the process, and with a hope that the subject of fatty degeneration (even if by this method not elucidated) may have its study placed on a broad basis by subjecting its tenets to the tests of systematized experiment and observation!

The morphological study of the results of diseased action might also be undertaken. The differences that obtain between normal individuals and those of the subjects of hereditary disease must be of importance to the anatomist and the pathologist.

The variations in the forms of the bones, as found in medical museums, are of a character that suggest their relation to inherited causes. Every clinical observer has noted the peculiar shape of the chest in families in which pulmonary phthisis is hereditary, even though the special tuberculous deposits are absent in some of its members. The clubbing of the finger-nails is a sign of the same disposition. Some writers, indeed, claim that in this class of subjects a special arrangement of the fibers of the pneumogastric nerve exists. Are these and similar morphological characters susceptible of being also gathered so as to contribute to the discussion of the transmission of acquired characters? Are not opportunities here presented for the medically-trained biologist to study the subject of heredity in a line so important, and, alas! with material so abundant? Other hereditary diseases, such as struma, syphilis, and gout, are less strongly marked than is the tuberculous, but even on this obscure horizon landmarks are detected that are of sufficient definiteness to guide the observer to well-defined plans of study. The animals of zoological gardens exhibit examples of acquired struma, the effects of which more especially distinguish the skeleton. Can any of these characteristics be transmitted? How would the skeleton of a tiger, let us say, born in captivity in the third and fourth generation differ from that of a feral type? After what manner may one expect taxonomic characters modified in these generations of prisoners?

The nature of malignant growths, it is not improbable, would find a solution in a line of research based upon a similar proposition. What proportions of malignant growths, such as the sarcomata, are met with in the feral state of quadrupeds as compared with those in the domesticated or the captive state? Can experiments be devised by which we may expect to cause these growths to appear by creating the favoring conditions? Can we study the genesis of the sarcomata to better advantage than has hitherto been done, by outlining the biography, the lineage, and to some extent possibly the destiny, of these tumors, by applying to them experimental methods of research?

Medically-trained men are not apt to become pure morphologists. The underlying thought is of *function* through which *structure* is modified. In its best sense, therefore, Physiological Anatomy is the branch of science that would be most developed. Let us suppose that John Hunter had lived in 1891, and had essayed his work by all the aids of modern science, and had undertaken a plan of investigation for the continuation of his labors: might he not have accepted some such scheme as I have here feebly attempted to portray? With the admiration we feel for his genius, let us not only have Hunterian orations, but in each medical center a Hunterian laboratory and a Hunterian museum.

"I am so utterly opposed to those cloud-builders who would divorce physiology from anatomy," says Haller, "that I am persuaded that we know scarcely anything of physiology that is not learned through anatomy." (Quoted from R. Cresson Stiles' *Life and Doctrines of Haller*, New York, 1867.)

In Solomon's House in the *New Atlantis*, in which Bacon essayed a scheme for intellectual advancement, we read of "parks and enclosures of all sorts of beasts and birds, which we use not only for view or rareness, but likewise for dissection and trials, that thereby we may take light what may be wrought upon the body of man; we have also particular pools where we make trials upon fishes, as we have said before of beasts and birds."

I hear objections that this scheme is visionary and impracticable. How is the money to be obtained by which it can be rendered feasible? Where is the teaching-force to be recruited? My answer is that if the need of establishing such a course be acknowledged, the accomplishment of the end in view is no more difficult than in any other branch of pure science. A few years ago the establishment of seaside laboratories would have been thought chimerical. Now they are assured successes.

If I am told the results obtained will appeal to but few, I reply that important projects must be supported in proportion as they so appeal, until such time as they shall have proved their right to exist.

